# Influence of age and gender on gait kinematics of pelvis and hip in healthy adults aged 19–60 years

**DOI:** 10.3389/fbioe.2024.1515583

**Published:** 2025-01-10

**Authors:** Rajani Mullerpatan, Triveni Shetty, Bela Agarwal

**Affiliations:** MGM Centre of Human Movement Science, MGM School of Physiotherapy, MGM Institute of Health Sciences, Navi Mumbai, India

**Keywords:** walking pattern, loading, gait biomechanics, indian population, sex-difference

## Abstract

**Purpose:**

Pelvic and hip motion are pivotal in maintaining postural control and energy efficient gait. An insight into influence of age and gender on the coupled motion of hip and pelvis in gait-cycle will guide clinical rehabilitation strategies and pertinent technology-design for specific age-groups. Therefore, present study evaluated pelvic and hip-joint gait kinematics in healthy females and males across adult-hood.

**Methods:**

Following signed-informed consent, pelvic and hip kinematics in 3-planes during stance-phase of gait were measured using 12-camera motion system and 2 force-plates, in 200 healthy Indian female and male volunteers (19–60years) stratified into 4-groups (19–30 years; 31–40 years; 41–50years; 51–60 years).

**Results:**

With advancing age, males and females demonstrated a gradual rise in hip adduction (p < 0.01) in coronal plane. Sagittal plane pelvic and hip kinematics did not change with advancing age among males whereas females above 30 years Demonstrated greater pelvic drop (49%), pelvic tilt (35%) and hip adduction (69%) compared to females below 30 years (p < 0.01). In comparison to males, females demonstrated greater peak anterior pelvic tilt (32%), greater pelvic hike (28%) and protraction (28%) in 50–60 years age-group (p < 0.05). Females across all age-groups demonstrated greater hip adduction compared to males (p < 0.05).

**Conclusion:**

Present findings add age and gender characterized gait-kinematics data of healthy adults from the most populous country to the existing 3-D data of gait from different populations. Clinicians and engineers, can leverage this knowledge of changing gait kinematics of healthy adults to design specific therapeutic strategies for aging men and women to optimize gait kinematics and advance design and development of locomotor technology suitable for people with rehabilitation needs across the globe.

## 1 Introduction

The coupled motion between pelvis and hip joint in all three planes assumes a pivotal role in human locomotion by the virtue of the mechanical position of pelvis and hip in the human body in erect antigravity postures (Smith et al., 2002). Pelvic motion optimizes the centre of mass (CoM) displacement resulting in an energy-efficient gait; whereas the hip joint is responsible for body-weight transfer as it forms the mechanical link between trunk and lower-extremity i.e., locomotor segment (Smith et al., 2002; Lewis et al., 2017). Therefore, the pelvis and hip motion are pivotal in postural control and energy cost of gait.

Joint motion of the hip and pelvis is influenced by various factors, including aging and pathological conditions (Hsu et al., 2003). While gait deviations due to pathological conditions are well-documented (Hesse et al., 1997; Hsu et al., 2003), the impact of physiological changes associated with healthy aging on pelvis and hip kinematics remains less understood. Aging brings about changes in muscle strength, joint flexibility, and neuromuscular control, all of which can affect gait biomechanics. Additionally, morphological differences between males and females, such as variations in pelvic anatomy and hip joint alignment, can further influence pelvis and hip motion during walking (Smith et al., 2002). Understanding these factors is essential to delineate the biomechanical changes in gait associated with normal aging (Smith et al., 2002).

Pelvic bone varies in morphological characteristics between females and males (Lewis et al., 2017). Female pelvis is wider and broader, with less prominent ischial spines than male pelvis; whereas the male pelvis presents longer, more curved sacrum, and a narrower subpubic arch (Smith et al., 2002). These variations in pelvic morphology are reflected in gender differences in kinematics and kinetics of walking (Hurd et al., 2004; Boyer et al., 2008; Chumanov et al., 2008; NiggKET et al., 2010; Ko et al., 2011). Previous researchers have hypothesised that broader pelvis in females may result in increased energy expenditure during gait; however, these assertions remain unsubstantiated empirically (Warrener et al., 2015). Conversely, greater pelvic obliquity in women compared to men, may reduce vertical COM displacement and thereby conserve energy expenditure during walking (Smith et al., 2002).

Nevertheless, variation in kinematics of pelvic motion between males and females are largely attributed to the broader pelvic structure in females. Smith et al. (2002). previous quantitative studies have revealed similarities in joint motion patterns and kinetic profiles between genders while walking at comparable velocities (Chatterley et al., 2007; Bruening et al., 2015). It has also been observed that women tend to walk at significantly higher cadence and shorter step length than men, and when adjusted for stature, women exhibit similar step length (Bruening et al., 2015).

Similarly, age has been shown to influence human gait because of the anatomical and physiological changes occurring in the neuro-musculo-skeletal and cardio-pulmonary systems (Oberg et al., 1994). Several studies have reported gait characteristics in older adults. However, most of these studies have reported difference in gait kinematics between younger and older adults; whereas the influence of age across stratified age groups in adulthood remains unclear (Grunte et al., 2010).

Additionally, apart from age and gender, ethnic background has been recognized as a significant factor which has an influence on body proportion and body composition (Kagawa et al., 2007). Anthropometric characteristics vary among various ethnic groups, particularly within Southeast Asia, wherein populations from adjacent countries present distinct body characteristics. Similarly, individuals from historically interconnected ethnic groups within East Asia also present variations in anthropometric traits (Kagawa et al., 2007).

Gait parameters are also known to be influenced by ethnicity; because of variation in anthropometric characteristics and variation in body postures influenced by cultural practices in daily life and environmental factors. For, e.g., daily living activities in Asian continent involve a range of postures adopted at ground level, which cross all three planes such as cross-legged sitting, squatting, low seating, squatting, kneeling with plantar-flexed ankle and kneeling with dorsi-flexed ankle and other high flexion postures (Ganokroj et al., 2021). Currently, a substantial volume of literature is available on gait patterns from the United States, Europe, Brazil and some information from South East Asia and Korea (Oberg et al., 1994; Chatterley et al., 2007; Kagawa et al., 2007; Pietraszewski et al., 2012). However, the evidence available from the most populous continent characterized by substantial cultural diversity, i.e., Asia is limited.

Therefore, present study was designed to understand how age and gender influence pelvic and hip kinematics during gait in the three planes of motion; in healthy Indian adults across age groups (19–60 years). Greater understanding of influence of age and gender on pelvic and hip kinematics in gait, across age-groups between males and females in different population groups will guide clinical rehabilitation strategies for locomotion and pertinent technology-design for specific age groups and gender.

## 2 Methodology

Present cross-sectional study was approved by the Institutional Ethical Review Committee (IERC: MGM/DCH/IEC/11/20) at MGM Centre of Human Movement Science, Navi Mumbai, India.

### 2.1 Participants

Two hundred healthy adult volunteers aged 19–60 years Were evaluated following signed-informed consent. The participants were stratified into 4 groups based on the age; 19–30 years, 31–40 years, 41–50 years, 51–60 years (Prince et al., 1997; Hollman et al., 2007). Volunteers presenting with diagnosed neuro-musculoskeletal disorders, traumatic injuries, developmental disorders, cognitive or psychiatric disorders, uncorrected visual defects, active illness, acute exacerbation of respiratory conditions or history of cardiac or respiratory dysfunction, uncontrolled diabetes (random blood glucose levels more than 200 mg/dL or below 60 mg/dL), uncontrolled hypertension and pregnant women were excluded. The BMI of all participants ranged within normal to overweight BMI range (22.3–26.8 kg/m^2^). None of the participants were obese or none of the participants in 50–60 years Presented with history of falls or fear of falls.

### 2.2 Procedure

Two hundred healthy participants aged between 19 and 60 years (98 males; 102 females) were evaluated to measure pelvis and hip kinematics during stance phase of gait using 12 camera Vicon Motion System (Oxford, United Kingdom) and 3 AMTI force plates (Advanced Mechanical Technology, Inc., Watertown, MA, United States). A Vicon Nexus motion capture system (Oxford Metrics Ltd., United Kingdom) with 12 infrared MX cameras (Bonita 10) 240 fps tracked the three-dimensional trajectories of reflective markers placed on the skin. Markers of 14 mm diameter were used to reduce crossover and merging. Each trial was examined for merge or crossover of marker trajectories. Gait trials were captured at a frequency of 100 Hz. Anthropometric data including body height, body mass, leg length, ankle width, knee width, inter ASIS distance, elbow width, hand thickness and wrist width was measured for each participant and entered into the software for calibrating the participant with the existing Vicon skeleton (vsk).

Each participant walked barefoot at a self-selected speed along a 10 m walkway. Mid-gait data were processed for further analyses. A minimum of 3 walk trials were recorded with each foot striking single force plate. Thirty-nine reflective markers were placed according to Plug-In Gait model (VICON Motion System, Oxford, United Kingdom). Data were filtered using Butterworth filter at a frequency of 6 Hz for trajectory data and 10 Hz for analogue data. Data were processed further using NEXUS 2.6 software (VICON Motion System, Oxford, United Kingdom) and joint angles were computed for further analysis. The Vicon analysis system has a system error of less than 2 mm. Vicon equipment and software can provide dynamic measurements down to 0.017 mm.

The primary kinematic variables of interest were extracted after processing the trials. Pelvic protraction is defined as the forward rotation of the pelvis in the transverse plane, while pelvic retraction is its backward rotation. These movements are counter-movements that occur alternately; when the right side of the pelvis is in protraction, the left side is simultaneously in retraction, and *vice versa*. Additional pelvic kinematics included pelvic tilt (anterior and posterior tilt) and pelvic obliquity (upward and downward movement in the frontal plane). Hip joint kinematics were also analysed, including hip flexion-extension, hip abduction-adduction, and hip internal-external rotation.

Statistical analyses were performed using SPSS (version 20.0). The level of significance was set at p ≤ 0.05 and 95% confidence interval were used for all statistical comparisons. Normality of distribution was ascertained and measures of central tendency and dispersion were calculated and reported as mean and standard deviation. Within-group comparison between males and females was performed using independent Student’s t-test. One-way analysis of variance (ANOVA) was used to study difference across age with age as an independent variable (factor). The linear contrast was applied to observe linear trend in pelvis and hip kinematic variables across age groups.

## 3 Results

The present study reported pelvis and hip kinematics of 200 healthy adults. The demographic characteristics are presented in Table 1. Male participants walked with a mean cadence of 109 (12.5) steps/min, and an average walking speed of 1.18 (0.15) m/sec. Female participants walked with a mean cadence of 115 (9.9) steps/min, with an average walking speed of 1.16 (0.16) m/sec.

**TABLE 1:** Demographic and anthropometric characteristics of males and females across age-groups.

	19–30 years (n = 62) Mean (SD)		31–40 years (n = 51) Mean (SD)		41–50 years n = 53 Mean (SD)		51–60 years n = 34 Mean (SD)	
	Male (n = 26)	Female (n = 36)	Male (n = 27)	Female (n = 24)	Male (n = 27)	Female (n = 26)	Male (n = 18)	Female (n = 16)
Age (year)	22.6 (3.1)	21.8 (1.9)	34.2 (2.8)	33.8 (2.9)	42.5 (2.7)	45.2 (2.9)	54.8 (2.9)	54.2 (2.7)
Inter ASIS (cm)	25.8 (0.2)	26.8 (0.4)	29.7 (0.37)	32.0 (0.41)	2.98 (0.39)	32.2 (0.24)	2.96 (0.25)	32.6 (23.1)
Height (m)	1.56 (4.5)	1.58 (1.8)	1.65 (6.8)	1.55 (4.5)	1.59 (3.7)	1.56 (2.8)	1.59 (1.9)	1.55 (0.55)
Weight (kg)	66.7 (10.4)	60.27 (10.4)	66.1 (11.8)	58 (10.6)	68.1 (9.8)	59 (10.3)	71.6 (5.8)	60.2 (6.5)
Cadence (steps/min)	109. (11)	110 (4.7)	106 (10.9)	113 (10.9)	112 (10.4)	110 (12.2)	105 (8.7)	108 (7.99)
Leg length (m)	8.84 (0.83)	8.73 (0.70)	8.96 (0.57)	8.70 (0.48)	8.76 (0.67)	8.36 (0.55)	8.92 (0.67)	8.65 (0.5)
Walking speed (m/s)	1.17 (0.25)	1.16 (0.4)	1.15 (0.3)	1.14 (0.34)	1.19 (0.22)	1.08 (0.27)	1.07 (0.1)	1.05 (0.15)
Centre of Mass (CoM) (mm)	35.3 (8.5)	31.7 (5.4)	39.9 (5.8)	32.15 (5.1)	40.9 (10.8)	33.12 (8.3)	41.9 (7.9)	34.9 (7.19)

Pelvis and hip joint kinematics across the age groups were normally distributed (p = 0.2) and did not differ between right and left side (p > 0.05) during gait instances. Hence, parametric tests were used and variables measured on one side, i.e., right lower extremity were considered for further description of gait variables.

### 3.1 Kinematic and kinetic variables

In the age group of 19–40 years, peak anterior and posterior pelvic tilt during stance phase of gait was not different between males and females. Females in 40–50 years age group demonstrated 2.2° greater peak anterior pelvic tilt than males; whereas females in the age group of 51–60 years demonstrated 4.5° greater anterior pelvic tilt compared to males (p < 0.01). These findings suggest that the gender-related variation in anterior pelvic tilt during walking was obvious from 40 year to 60 year (Table 2).

**TABLE 2:** Pelvis and hip kinematics of males and females during stance phase of gait across different age groups.

Gait variables in stance	19–30 years		31–40 years		41–50 years		51–60 years		Males	Females
	Mean difference between males and females	Student’s t-test p-value	Mean difference between males and females	Student’s t-test p-value	Mean difference between males and females	Student’s t-test p-value	Mean difference between males and females	Student’s t-test p-value	ANOVA p-value	ANOVA p-value
Peak anterior pelvic tilt (^0^)	2	0.1	3	0.1	2.2	0.01	4.4	0.05	0.1	<0.01
Peak posterior pelvic tilt (^0^)	1.9	0.4	3.8	0.4	1.8	0.2	2.3	0.2	0.3	<0.01
Peak pelvic hike (^0^)	0.45	0.4	1.5	0.08	1.8	0.01	1.9	0.01	0.4	<0.01
Peak pelvic drop (^0^)	1.9	0.1	3.4	0.3	1.18	0.3	2.7	0.1	0.1	<0.01
Peak pelvic protraction (^0^)	0	0.9	0.6	0.4	0.9	0.3	2.2	0.05	0.4	<0.01
Peak pelvic retraction (^0^)	0.5	0.1	0.1	0.1	0.1	0.4	1.3	0.3	0.3	<0.01
Peak hip Flexion (^0^)	0.6	0.2	0.6	0.8	1.84	0.4	2.3	0.1	0.5	0.4
Peak Hip extension (^0^)	1.66	0.2	2.1	0.8	0.4	0.1	2.4	0.8	0.1	0.3
Peak hip abduction (^0^)	1.8	0.01	4.5	0.03	0.2	0.1	0.1	0.83	<0.01	<0.01
Peak Hip Adduction (^0^)	3.4	0.01	8.6	0.02	3.5	0.01	3.9	0.01	<0.01	<0.01
Peak hip Internal rotation (^0^)	2.6	0.1	4.1	0.3	8.2	0.8	2.1	0.7	0.1	0.2
Lower extremity joint moments										
Hip flexor moment	0.05	0.6	0.03	0.8	0.01	0.9	0.1	0.83	<0.05	0.2
Hip extensor moment	0.02	0.9	0.04	0.7	0.07	0.09	0.3	0.2	<0.01	<0.01
Hip Adductor moment	0.03	0.9	0.09	0.4	0.1	0.9	0.3	0.1	<0.01	<0.01

Student’s t-test: p < 0.05.

ANOVA: p < 0.01.

**FIGURE 1:**
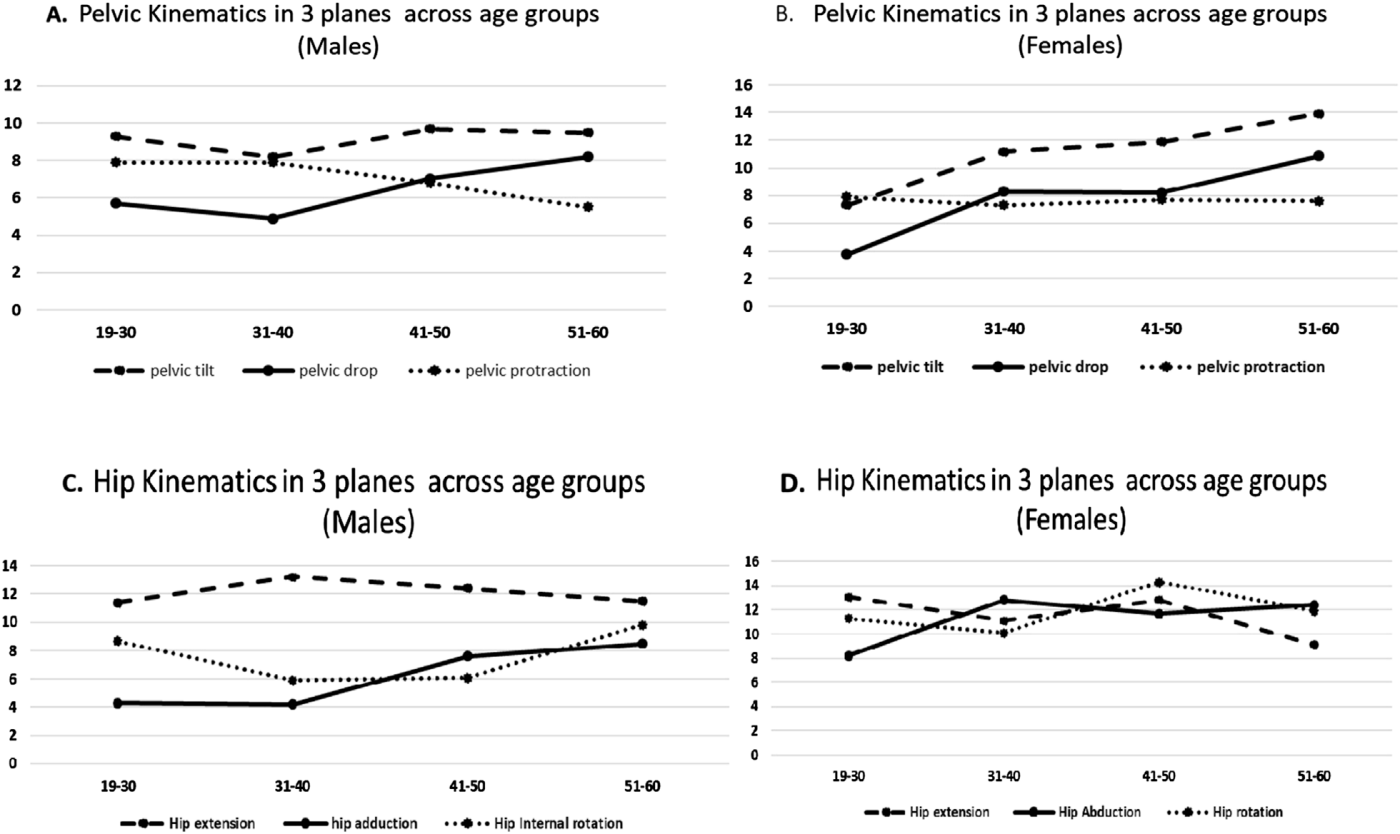
**(A)** Pelvic kinematics in 3 planes across age groups (males). **(B)** Pelvic kinematics in 3 planes across age groups (females). **(C)** Hip kinematics in 3 planes across age groups (males). **(D)** Hip kinematics in 3 planes across age groups (females).

A linear rise in anterior pelvic tilt was observed in females across the age range of 19–60years (p < 0.05). Males maintained the anterior pelvic tilt with advancing age; whereas females demonstrated greater difference in magnitude of peak anterior pelvic tilt across age groups. The younger females in 19–30 years age-group yr. Demonstrated least anterior pelvic tilt, whereas the oldest age group between 51 and 60 years, demonstrated 6.7° greater anterior tilt during stance phase of gait cycle (Figure 1).

Sagittal plane kinematic analysis of hip joint revealed that males and females in 19–60 years age group demonstrated similar magnitude of peak hip flexion and extension during stance phase of gait. However, with advancing age, the difference in magnitude of peak hip flexion at initial contact was 6.3° greater among females in 51–60 years age group compared to females in 19–30 years age group (p < 0.01).

In the frontal plane, females above 40 years Demonstrated 1.9° greater pelvic hike compared to males during stance (p < 0.05). However, the observed difference in pelvic obliquity between males and females was not statistically significant in 19–40 years age group (p > 0.05). With advancing age, male participants did not demonstrate difference in the magnitude of peak pelvic drop during stance phase of gait (p > 0.01); however, females demonstrated 7.1° greater pelvic drop during stance phase of gait (p < 0.01).

**FIGURE 2:**
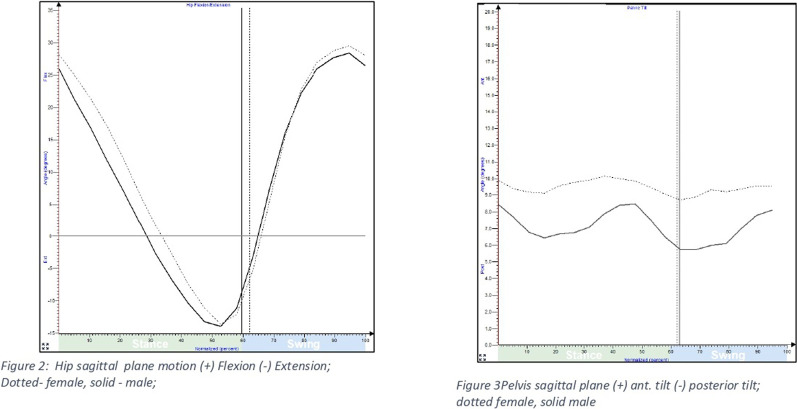
Hip and pelvic sagittal plane motion during gait among males and females.

Frontal plane hip kinematics revealed that males in 19–40 years age group demonstrated greater peak hip abduction compared to females during terminal stance (p < 0.05). However, females demonstrated greater peak hip adduction during mid-stance compared to males across all age groups, i.e. 19–60 years (p < 0.05). (Figures 2A, B).

Peak hip abduction demonstrated a linear decline with advancing age amongst both male and female participants (p < 0.01). The decline was greater in males (5.7° lower hip abduction) than females (3.7° lower hip abduction) in comparison to the younger participants. The magnitude of peak hip adduction increased by 4° in females above 30 yr; whereas males demonstrated an increase of 3.5° in peak hip adduction above 40 yr (p < 0.01).

**FIGURE 3:**
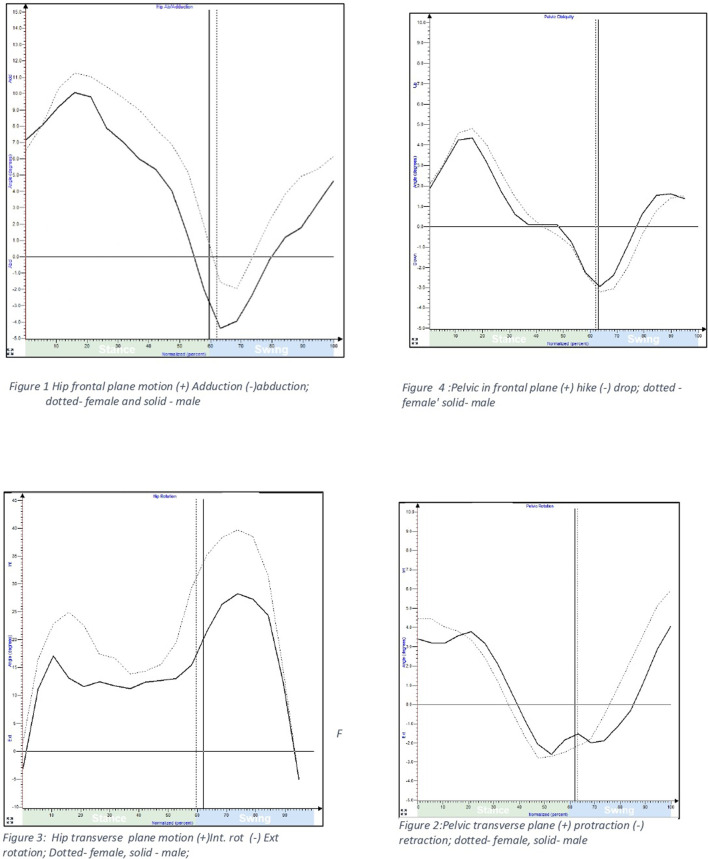
Hip and pelvic motion in frontal and transverse plane during gait among males and females.

In the transverse plane, older females in 50–60 years age group presented 2.1° greater average peak pelvic protraction than males. (p < 0.05); whereas no gender difference was observed in the other age groups between 19 and 50 years age group (Figure 3).

Transverse plane hip kinematics revealed greater average values of peak hip internal rotation in females compared to males in all age groups, although the difference was non-significant. The variability as measured by standard deviation was small.

### 3.2 Kinetic variables

The hip joint moments in the sagittal plane were consistent across the 19–50 years age range for both genders. However, individuals between 50 and 60 years, irrespective of gender, demonstrated a 35% greater hip flexor and extensor moment during the stance phase of gait compared to those aged 19–30 years (p < 0.05).

**FIGURE 4:**
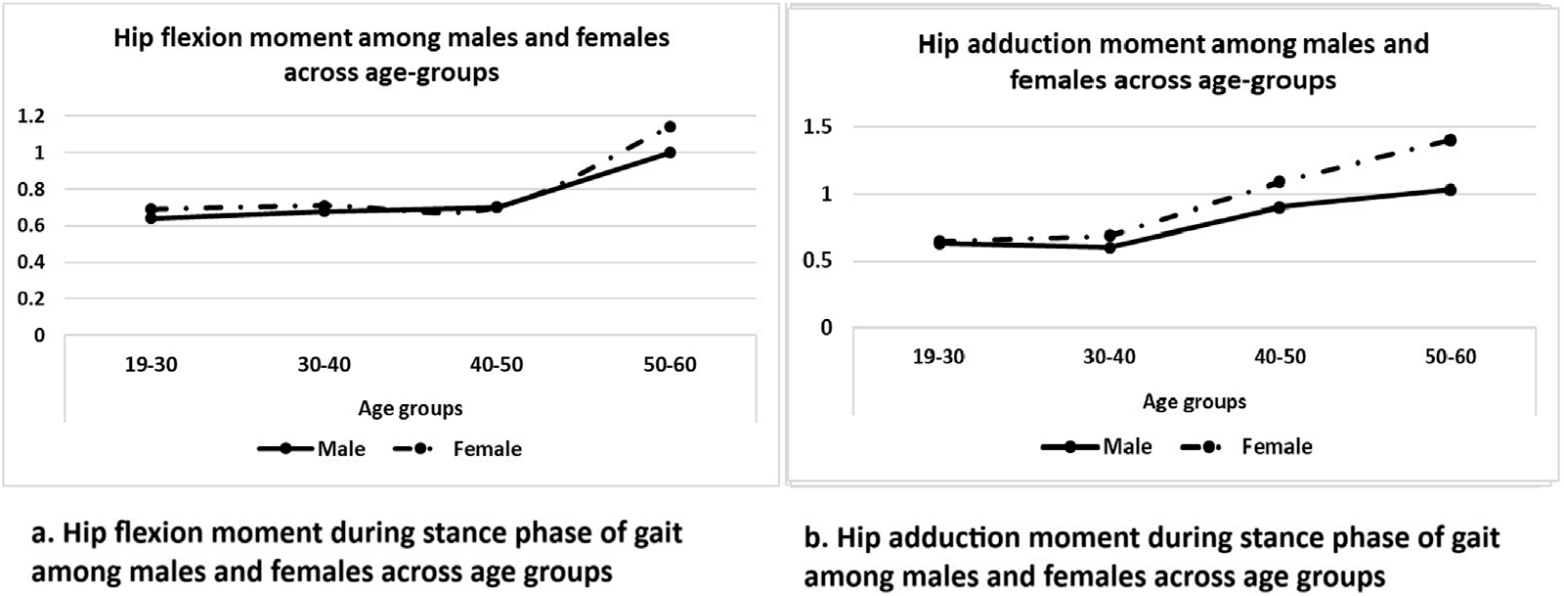
**(A,B)** Hip flexion and adduction moment during stance phase of gait among males and females across age group respectively.

In the frontal plane, hip joint moments were similar between males and females across the 19–40 years age range. However, the hip adductor moment increased significantly with age. Individuals aged 40–50 years exhibited a 37% higher hip adductor moment (p < 0.01), while those aged 50–60 years demonstrated a 60% greater hip adductor moment (p < 0.01) during the stance phase compared to individuals aged 19–40 years (Figures 4A, B).

## 4 Discussion

Gait is considered as the sixth vital sign, which is widely used to assess physical function, health-related quality of life, and health status in people with movement dysfunction and the elderly (Ireland et al., 2003). Evaluation of kinematic characteristics of gait, provides important information necessary to guide neuro-musculoskeletal rehabilitation and rehabilitation engineering. Application of precise information on gait characteristics in clinical rehabilitation and rehabilitation engineering for various population groups worldwide, demands robust information on gait parameters studied in different population groups.

Present study reports age and gender differences in pelvis and hip kinematics and kinetics during gait in healthy adults from Indian population.

During gait, the pelvis moves in three planes to produce smooth and efficient motion. Theoretically, pelvic motion in frontal and transverse plane are known to minimize vertical and horizontal displacement of the CoM (centre of mass) (Lewis et al., 2017). Additionally, morphological variation in male and female pelvis also influences the displacement of CoM Wider pelvis and deeper acetabular socket among females is speculated to shift the CoM medially towards the hip joint centre; thereby reducing the distance of the moment arm of hip abductor muscles (Smith et al., 2002).

Wider pelvis and shorter moment arm of hip abductor muscles demands greater abductor muscle force to stabilize the pelvis during gait in females. This gender-related gait variation caused by the specific pelvic morphology of the females is known (Delp and Maloney, 1993).

Previous studies reported morphological and anatomical differences in characteristics of female pelvis that may facilitate an energy efficient gait despite subtle differences in pelvic motion (Lewis et al., 2017). Present findings demonstrated that females above 40 years Walked with 19%–32% anterior tilt, 28% greater pelvic hike compared to age-matched males; whereas females above 51 years Demonstrated 28% greater pelvic protraction compared to males. Secondly, females demonstrated a linear rise in anterior pelvic tilt with each decade across advancing age groups.

Older females aged 40–60 years Demonstrated 35% greater tilt and 49% greater pelvic hike compared to females younger than 40 yr. Analysis of hip kinematics in the frontal plane revealed that females demonstrated greater hip adduction compared to males; however, with advancing age there was a 38% rise in hip adduction after 30 years In females and 35% rise after 40 years In males. The early rise noted in the magnitude of frontal plane kinematics in females by 1 decade, compared to males can be attributed to the physiological changes occurring in the child bearing age of women above 30 years. These changes are often associated with muscle weakness, particularly in the trunk core muscles, hip abductors, and pelvic floor muscles (Ireland et al., 2003; Ferber et al., 2003).

The muscle volume in females is lower compared to males due to lower testosterone and increased percentage of fat mass due to oestrogen. Aging is known to reduce the muscle volume further. However, the effect of muscle loss may be more pronounced in females because the number of muscle fibres and muscle size is smaller in females compared to males (Ferber et al., 2003). Age related reduction in trunk core muscle strength increases the displacement of CoM and thereby reduces the stability of the pelvis and trunk (Ferber et al., 2003; Chevidikunnan et al., 2016). Thus, it could be explained that reduction in trunk core muscle strength can translate in increased anterior and posterior pelvic tilt (Phinyomark et al., 2014).

The pelvis and hip exhibit tri-planar motion and display inter-related coupled movement patterns during gait (Lewis et al., 2017). In the sagittal plane, the hip joint demonstrates a relatively larger arc of range of motion; yet the age and gender-related differences are comparatively modest, primarily due to lower gluteus maximus and iliopsoas muscle demands. Conversely, in the coronal plane, the hip joint’s arc of range of motion is smaller; yet the age and gender related differences are more pronounced, largely because of higher demand in hip abductor muscle strength essential for postural stability in coronal plane (Chatterley et al., 2007).

With reference to pelvic motion, particularly pelvic obliquity and pelvic rotation, females generally exhibit a broader arc of motion compared to males, except in the case of pelvic tilt, where young males tend to display a wider range of motion. These observations underscore the multifaceted dynamics of pelvic and hip movements, which vary across genders and planes of motion (Lewis et al., 2017).

Additionally, results of the present study revealed age-related increases in hip joint moments in both the sagittal and frontal planes, highlighting biomechanical adaptations with advancing age that are distinct in their presentation. The hip joint moments in the sagittal plane remained consistent across individuals aged 19–50 years for both genders. However, adults aged 50–60 years exhibited 35% greater hip flexor and extensor moments during the stance phase of gait (p < 0.05) compared to individuals aged 19–30 years. The greater sagittal plane moment reflects compensation mechanisms adopted by the body to maintain stability and propel the body forward during gait. These compensatory mechanisms could be attributed to age-related decline in muscle strength, particularly in the hip extensors (gluteus maximus) and flexors (iliopsoas) (Boyer and Nigg, 2006; Kulmala et al., 2016).

In the frontal plane, hip adductor moment was 37% greater in individuals aged 40–50 years and 60% greater in individuals aged 50–60 years compared to individuals aged 19–40 years. These findings align with previous studies showing that advancing age places greater reliance on hip abductors for frontal plane stability during the stance phase (Landry et al., 2007; Foucher et al., 2010). The increased adductor moment could be attributed to age-related reductions in strength of hip abductor muscles, which is critical for stabilizing the pelvis and maintaining frontal plane balance during single-leg stance phases of gait (Mademli and Arampatzis, 2008).

Present findings concur with previous studies, which have consistently observed variations in frontal plane hip joint angles among young, middle-aged, and older healthy males and females during walking (Chatterley et al., 2007; Jang and Kim, 2017). It has been postulated that increased frontal plane hip motion, along with weak hip abductor muscles, may add to the increased predisposition of healthy females to musculoskeletal injuries like patellofemoral pain or iliotibial band syndrome with advancing age (Prince et al., 1997), in comparison to age-matched males. This can be attributed to the shift of CoM away from the hip joint, thereby increasing knee adductor moment, further affecting the activation of hip abductor muscle and increasing the muscular demand of quadriceps muscle (Hemmerich et al., 2006).

The pelvic kinematics in sagittal, frontal, and transverse planes among Indian population in present study demonstrated variation with reference to Caucasian population reported in the literature (Bruening et al., 2015). It is observed that the average values of pelvic kinematics among Young Indian males and females aged 19–30 years Was 40% in sagittal plane, 45%–48% greater in frontal plane and 40% lesser in transverse plane than the average values of the Caucasian men and women reported in a previous study (Bruening et al., 2015).

Variations in gait in different ethnic groups could be attributed to various intrinsic and extrinsic factors. Extrinsic factors such as methods used to capture gait can introduce minor variations in gait variables. Although both studies used optical camera systems to capture gait, the testing protocol and processing software’s were different. Present study utilized optical motion capture systems with an accuracy of 1 mm to determine marker position in space, which is lower than the error caused by the use of non-optical systems to capture gait kinematics and kinetics (Pietraszewski et al., 2012).

Intrinsic factors contributing to differences in sagittal and frontal plane pelvic kinematics can be attributed to exposure to various floor level postures adopted by Indian population for several activities of daily living such as squatting, cross-legged sitting and kneel sitting (Hemmerich et al., 2006). Most Indians are exposed to these floor level high flexion postures since childhood for a wide range of activities such as eating meals, ceremonial functions, social gatherings, prayers, performing household chores and occupational activities. These body postures expose the lower extremity joints to a combination of sagittal, frontal and transverse plane movements with extreme range of hip, knee flexion and pelvis tilt.

Participants in the current study represented urban and semi-urban Indian population which engage in a larger quantum of high-flexion postures. Floor level sitting requires a coordinated effort among the hip, knee, and ankle joints, particularly with regards to hip range of motion (ROM) (Mademli and Arampatzis, 2008). Studies indicate that large quantum of exposure to high flexion postures may bring about structural adaptions in the joints which can affect joint motion with aging (Hollman et al., 2007). It has been observed that Asians tend to exhibit greater hip flexion and external rotation ROM angles compared to individuals from Western cultures during high-flexion activities, which could be attributed to cultural and lifestyle differences observed in daily living activities (Jang and Kim, 2017).

An understanding of joint kinematics may help to address therapeutic needs of people presenting with common musculoskeletal and neurological conditions such as low back pain, knee osteoarthritis and stroke, where greater alterations in gait are observed. Normative values of gait provide a reference for interpretation of pathological gait cycle and assist clinicians to set goals for targeted rehabilitation interventions for gait retraining and prescription of assistive devices.

Further, in the era of global citizens seeking care in different parts of the world, clinicians and engineers, can leverage the knowledge of variation in gait kinematics from the most populous country to guide design and development of locomotor technology suitable for people with rehabilitation needs. The global movement of people places a binding demand on healthcare providers to cater to specific needs of people to match the gaps within diverse clinical needs and provision of standard care through guidelines.

Limitations of study: Present study does not report age and gender related differences in gait kinematics among older people above 60 years And people with yr. BMI more than 25 kg/m^2^ hence the findings should be interpreted accordingly. Further studies exploring the influence of various intrinsic factors such as individual body segment lengths and nutrition, which directly and indirectly affect gait kinematics will add greater insights into age and gender-related differences in gait kinematics observed in different populations worldwide.

## 5 Conclusion

A steady rise in hip adduction in coronal plane was observed in males and females with advancing age. Females demonstrated greater pelvic drop coupled with increasing hip adduction. In comparison to males, females demonstrated greater peak anterior pelvic tilt, greater pelvic hike and protraction in 50–60 years age-group. Whereas females across all age-groups demonstrated greater hip adduction compared to males. Clinicians and engineers, can leverage this knowledge of age and gender related gait kinematics of healthy adults to design specific therapeutic strategies for aging men and women to optimize gait kinematics and guide design and development of locomotor technology suitable for people with rehabilitation needs across the globe.

## Data Availability

The original contributions presented in the study are included in the article/supplementary material, further inquiries can be directed to the corresponding author.
